# The evaluation of a hybrid biomechanical deformable registration method on a multistage physical phantom with reproducible deformation

**DOI:** 10.1186/s13014-018-1192-x

**Published:** 2018-12-04

**Authors:** An Qin, Dan Ionascu, Jian Liang, Xiao Han, Nicolette O’Connell, Di Yan

**Affiliations:** 1 Department of Radiation Oncology, Beaumont Health System, Royal Oak, MI USA; 2 Department of Radiation Oncology, College of Medicine, University of Cincinnati, Cincinnati, OH USA; 3 Elekta Inc., Maryland Heights, MO USA

**Keywords:** Deformable image registration, adaptive radiotherapy, IGRT, Biomechanical model, dose accumulation

## Abstract

**Background:**

Advanced clinical applications, such as dose accumulation and adaptive radiation therapy, require deformable image registration (DIR) algorithms capable of voxel-wise accurate mapping of treatment dose or functional imaging. By utilizing a multistage deformable phantom, the authors investigated scenarios where biomechanical refinement method (BM-DIR) may be better than the pure image intensity based deformable registration (IM-DIR).

**Methods:**

The authors developed a biomechanical-model based DIR refinement method (BM-DIR) to refine the deformable vector field (DVF) from any initial intensity-based DIR (IM-DIR). The BM-DIR method was quantitatively evaluated on a novel phantom capable of ten reproducible gradually-increasing deformation stages using the urethra tube as a surrogate. The internal DIR accuracy was inspected in term of the Dice similarity coefficient (DSC), Hausdorff and mean surface distance as defined in of the urethra structure inside the phantom and compared with that of the initial IM-DIR under various stages of deformation. Voxel-wise deformation vector discrepancy and Jacobian regularity were also inspected to evaluate the output DVFs. In addition to phantom, two pairs of Head&Neck patient MR images with expert-defined landmarks inside parotids were utilized to evaluate the BM-DIR accuracy with target registration error (TRE).

**Results:**

The DSC and surface distance measures of the inner urethra tube indicated the BM-DIR method can improve the internal DVF accuracy on masked MR images for the phases of a large degree of deformation. The smoother Jacobian distribution from the BM-DIR suggests more physically-plausible internal deformation. For H&N cancer patients, the BM-DIR improved the TRE from 0.339 cm to 0.210 cm for the landmarks inside parotid on the masked MR images.

**Conclusions:**

We have quantitatively demonstrated on a multi-stage physical phantom and limited patient data that the proposed BM-DIR can improve the accuracy inside solid organs with large deformation where distinctive image features are absent.

## Introduction

Deformable image registration (DIR) has become one of the key technologies that enable adaptive radiation therapy (ART), not only for the purposes of contour propagation, but also for dose warping [1–5], treatment response evaluation [6, 7] and 4D-inverse plan adaptation [8–10]. Most of the existing DIR methods are image intensity-based (IM-DIR) which utilize non-physical regularization [11]. Due to the intrinsic lack of proper physical modelling, they may generate unrealistic deformation especially when organs undergo large deformation and there are few distinctive image features available to follow [12–14]. While problematic internal deformation may not negatively impact contour propagation accuracy, it could lead to significant discrepancy when used to wrap dose or functional image [15–18].

A promising methodology of improving voxel-wise accuracy is by incorporating realistic biomechanical models. Finite-Element method (FEM) based biomechanical models were first introduced to register preoperative and intraoperative images to improve surgical accuracy [19, 20]. A biomechanical model was first proposed for treatment dose accumulation application [21]. A multi-organ biomechanical model has been introduced to simulate complex physiology process or treatment response [22]. However, biomechanical model based DIR (BM-DIR) methods, which need volume mesh construction, material property, and boundary condition assignment are more labour intensive and computation demanding than IM-DIR. Pure biomechanical modelling accuracy could be improved by incorporating image features matching. Recently, hybrid methods that take advantage of merits from both IM-DIR and physical modelling have been reported by several institutions. For instance, Zhong et al. use physical FEM model and boundary condition derived from intensity-based registration to further improve DIR accuracy in low-contrast region [23, 24]. Hybrid models were also proposed to improve lung DIR accuracy by incorporating IM-DIR or matching vessel tree features as boundary condition [25, 26].

A question arises regarding how to choose between IM-DIR and BM-DIR for a specific clinical task. In most clinical applications, IM-DIR methods most likely will continue to be the methods of choice since they are commercially widely available, fast with GPU acceleration, and produce decent contour-propagation results in most cases. For advanced applications that require voxel-wise accuracy, an image-biomechanical hybrid algorithm can be warranted. The additional computational efforts to use BM-DIR evidently depend on imaging modality, magnitude of deformation, and the richness of image features. Therefore, evaluation of its extra accuracy improvement over IM-DIR in the absence of ground truth is required. Proposals for accuracy-evaluation phantoms consisted in both physical [16, 27] and digital artificial-deformed phantoms [28–30]. Most of the previous studies were limited to single image modality and single deformation state.

In this work, we have developed a BM-DIR hybrid method based on a physical realistic model that can automatically refine the IM-DIR DVF inside designated regions. Furthermore, to determine the accuracy and proper situations of using the BM-DIR, we have evaluated it with a novel, multi-imaging modality soft tissue-equivalent deformable phantom and on real Head and Neck (H&N) patient MRI.

## Methods and materials

### Full automatic hybrid biomechanical DIR refinement (BM-DIR)

**Fig. 1:**
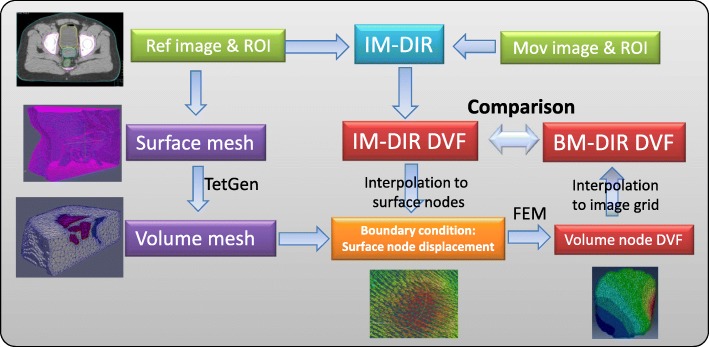
BM-DIR refinement workflow: the initial IM-DIR provides boundary condition for BM-DIR; volume mesh is generated from organ contour; FEM model is constructed to refine DVF

To correct for potential erroneous IM-DIR DVF inside regions of interest, a hybrid method of incorporating a physical model into pure image DIR has been developed. The specific implementation consists of steps like volume mesh generation, boundary condition assignment and DVF interpolation (depicted in Fig. 1).

#### Mesh generation

The quality of the volumetric mesh has a direct impact on the accuracy of FEM calculation [31]. To generate a high-quality tetrahedron mesh for human organs, a surface mesh is first generated on the reference image using the marching cube algorithm based on organ delineation from surrounding tissue. A multi-organ tetrahedral mesh is generated using TetGen [32]. The tetrahedron is chosen for its flexibility in representing various human organs, regardless of shape or topology and its capability of adaptive mesh refinement. The volume mesh can be generated offline to save computational time, including all ROIs that could potentially undergo large deformations through the treatment process. If any high-confidence feature points are detected either by an expert or the algorithm, they can be inserted into the nodes list during the mesh generation to facilitate a later boundary condition assignment. To ensure the generation of high-quality mesh, we fine-tuned the parameters of TetGen based on nine tetrahedron quality measures [33] for the typical critical organs in radiotherapy treatment. Furthermore, to achieve high interpolation accuracy, the average tetrahedron volume is chosen to be slightly smaller than that of the image voxel.

#### Boundary conditions

The contrast of organ surfaces generated from medical images is typically higher than the surrounding tissues, making possible to obtain contours with well-defined features. Based on that assumption, the surface node displacement is linearly interpolated from IM-DIR DVF and utilized as the boundary condition for the FEM deformation. A research version IM-DIR algorithm of a commercial DIR tool (ADMIRE1.12, Elekta Inc.) with GPU acceleration [34] was used for the initial DIR in this study with default setting in this study. The algorithm utilizes local correlation coefficient for the image similarity metric [35]. In this algorithm, the nonlinear deformation is computed in a coarse-to-fine fashion using a Gaussian pyramid representation. The final optimization objectives include image similarity, DVF regularization. The algorithm employed by ADMIRE has been reported and extensively evaluated on public dataset for multiple treatment sites [36–39] and in our institution for Head&Neck patients [40]. The core of the ADMIRE intra-patient DIR is a local-correlation-coefficient (LCC) based dense non-linear registration algorithm with a regularization term defined as L2 norm of first-order spatial derivative of the DVF. In addition, spatial Gaussian filter is used to regularize the intermediate DVF at each iteration and multi-resolution stage. However, like most other commercial DIR tools, we don’t have control over the type, weight or the parameter of the regularization. In some challenging clinical cases, when large deformation may happen due to treatment response or physiological processes (such as bladder filing or tumour regression), direct IM-DIR may fail to match the organ surface. The ADMIRE software provides an organ-constrained DIR function able to ensure the accurate surface matching. It should be noted that any DIR tool could be used as a substitute within the proposed BM-DIR framework as long as it can provide an accurate surface matching.

#### BM-DIR refinement

The BM-DIR is executed for each individual organ after IM-DIR. The biophysical organ models are generated using a tetrahedral mesh, surface nodes displacements, and organ-specific physical properties. A finite element solver package (ABAQUS, v6.14, Pawtucket, RI) was used to calculate the displacement of internal nodes. The organ-specific physical properties can be assigned to its respective tetrahedron mesh based on image intensity, however, our previous simulation has shown the uncertainty of material physical properties only has small impact on registration accuracy, especially for soft tissue organs [41]. For simplicity, uniform physical properties of Poisson ratio and Young’s moduli were assigned for the prostate phantom in this study. Solid soft-tissue organs physical deformation was described using a linear elastic model with the default setting of Poisson ratio 0.40 and Young’s moduli 0.27 MPa [42]. The refined DVF is generated by interpolating the displacements on internal nodes to the image grid by scattered linear interpolation. The time of the whole BM-DIR process, from the initial IM-DIR, mesh generation to final refined DVF, takes around 4–6 min for a typical prostate-size organ. The IM-DIR step takes about 1 min for a typical CT pair.

### Multi-modality tissue-equivalent phantom

**Fig. 2:**
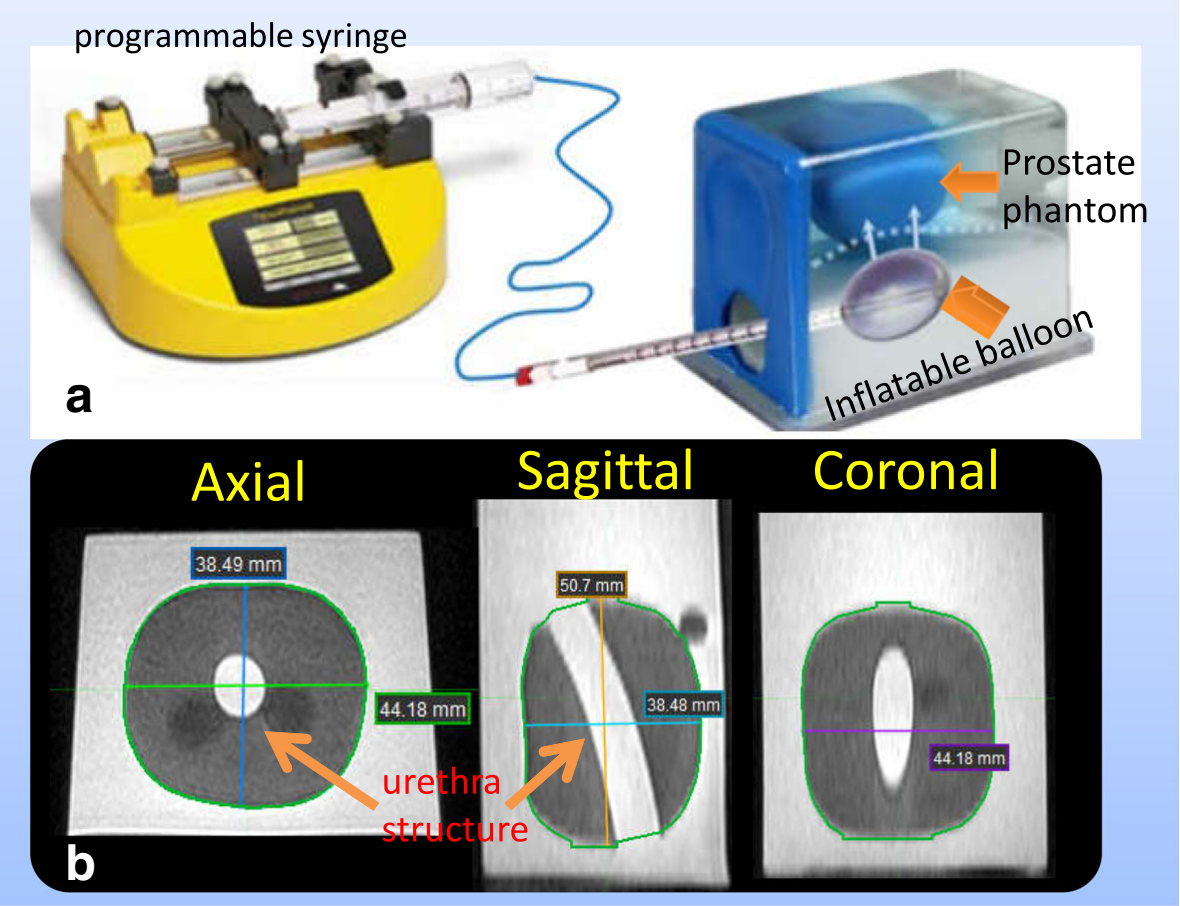
Multi-modality tissue-equivalent deformable phantom: **a** Phantom and Programmable syringe, **b** MR image of un-deformed phantom and its dimension

**Fig. 3:**
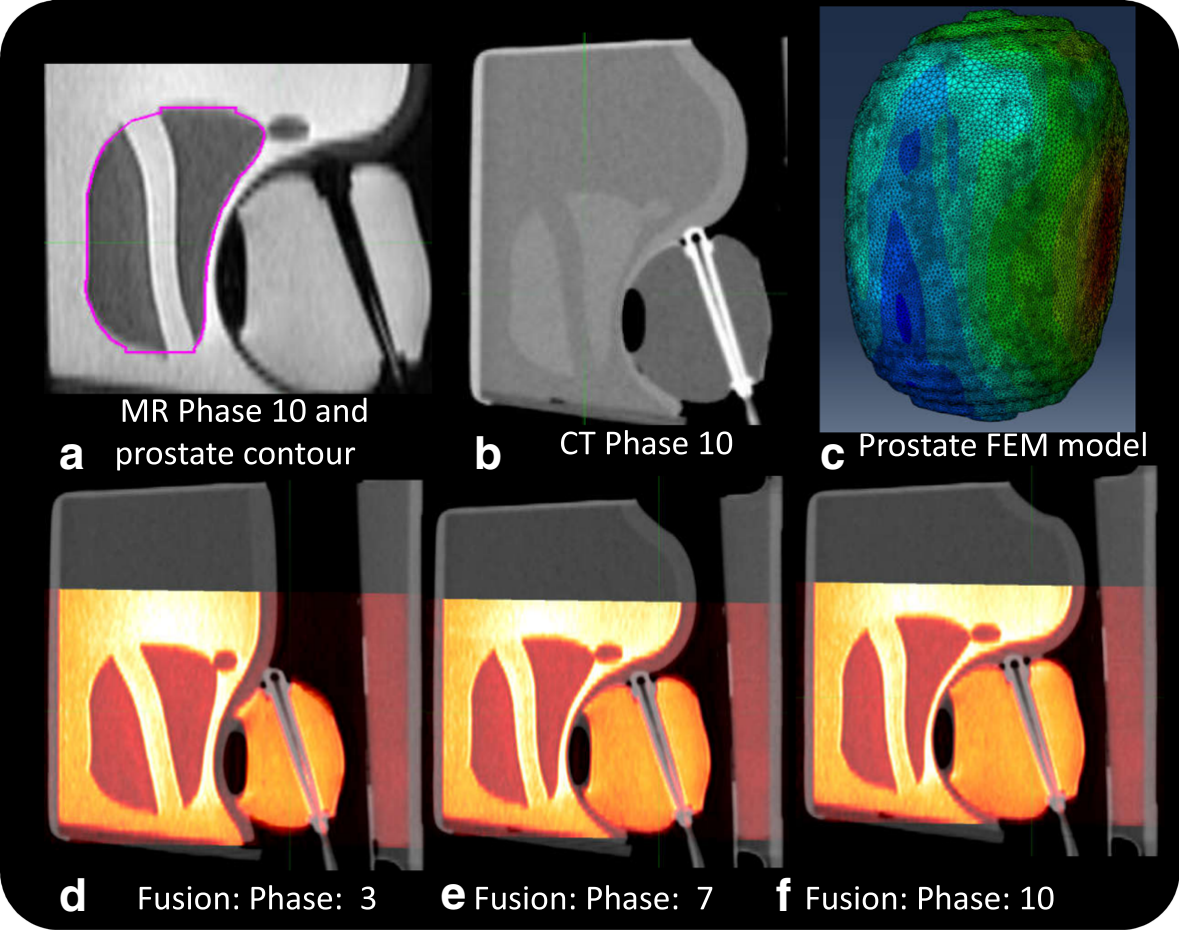
Deformed phantom (**a**) MR phase 10 with contour, **b** CT phase 10, **c** FEM model, **d-f** MR-CT Fusion: deformation phase 3,7,10

Generally, BM-DIR methods are more complicate to implement than pure IM-DIR. To investigate the proper situations where BM-DIR is more desirable, a soft-tissue equivalent phantom with reproducible and controllable deformation was developed [43]. Briefly, our phantom is based on a Ultrasound Prostate Training Phantom (CIRS, Norfolk, VA) -- a multi-modality phantom providing anatomical imaging contrast under CT, MRI, ultrasound and elastography similar to that of patient. The prostate along with structures simulating the rectal wall, seminal vesicles, perineal membrane and urethra is contained within clear acrylic container. In our deformable phantom, the prostate gland is programmatically deformed and displaced using an inflatable balloon controlled by a programmable syringe pusher as shown in Fig. 2(a). Ten different stages of deformation were created by controlling the syringe position and imaged under CT (Philips Brilliance Big Bore) and MR (Philips Ingenia 3.0 T Spine Echo, TR 2000 ms, TE 40 ms). The original resolution is 0.03, 0.03, 0.2 cm for MR and 0.05, 0.05, 0.1 cm for CT. Both the CT and MR images are resampled to the same resolution 0.05, 0.05, 0.2 cm before DIR to facilitate direct DVF comparison without interpolation uncertainty. Figure 2 shows the phantom setup and the un-deformed MR images (phase 0). The un-deformed linear dimensions are 4.42, 3.85 and 5.07 cm in anterior–posterior, lateral and craniocaudal directions, respectively. The tube structure simulating urethra shows sharp contrast on MR images and less contrast on CT images (Fig. 2 and Fig. 3). The MRI and CT of the most extreme-deformed stage (phase 10) is shown in the top row in Fig. 3. The fusion of CT and MR images on phase 3, 7, and 10 are shown in bottom row from left to right, where highly reproducible deformation states can be observed between the two imaging modalities. The contours of the prostate phantom and urethra structure were delineated on MR phase 0, and propagated to other phases by using ADMIRE, then reviewed and edited if necessary. The third set of images (Masked MR), was generated by overriding the inner voxels with the average intensity inside prostate contour (the green contour in Fig. 2b and magenta contour Fig. 3a). The purpose of generating the Masked MR is to emulate intensity-homogeneous soft-tissue organs as manifested on CT.

### Quantitative evaluation of BM-DIR on the multistage phantom

The IM-DIR and BM-DIR were performed between Phase 0 as reference and Phase *i* (*i* = 1 to 10) for CT, MR and masked MR with prostate contour as constrain, generating six types of DVFs for each phase. Since MR images had much better contrast inside prostate, the output DVFs were used to warp the MR Phase *i* images to fuse with MR phase 0 for a qualitative visual inspection. The tubular structure inside this prostate phantom --simulating the urethra-- has excellent contrast on MR image and can serve as surrogate for internal deformation accuracy. The urethra tube was deformed from phase *i* to phase 0 via different DVFs. The DSC, Hausdorff and mean surface distance as defined in [44] were calculated between the propagated and human-delineated urethra tube to evaluate the accuracy of internal DVF. The Jacobian determinant of DVFs from the MR and masked MR images were calculated and compared between IM-DIR and BM-DIR. In addition, the voxel-wise discrepancy between DVF from IM-DIR and BM-DIR inside the prostate phantom was evaluated on all ten phases. Target Registration Discrepancy (TRD) was calculated for every voxel inside the prostate phantom as the vector distance between the warped points generated using the IM-DIR and BM-DIR. The histogram of TRD was compared between different image types and deformation phases.

### Quantitative evaluation of BM-DIR on the patient data

**Fig. 4:**
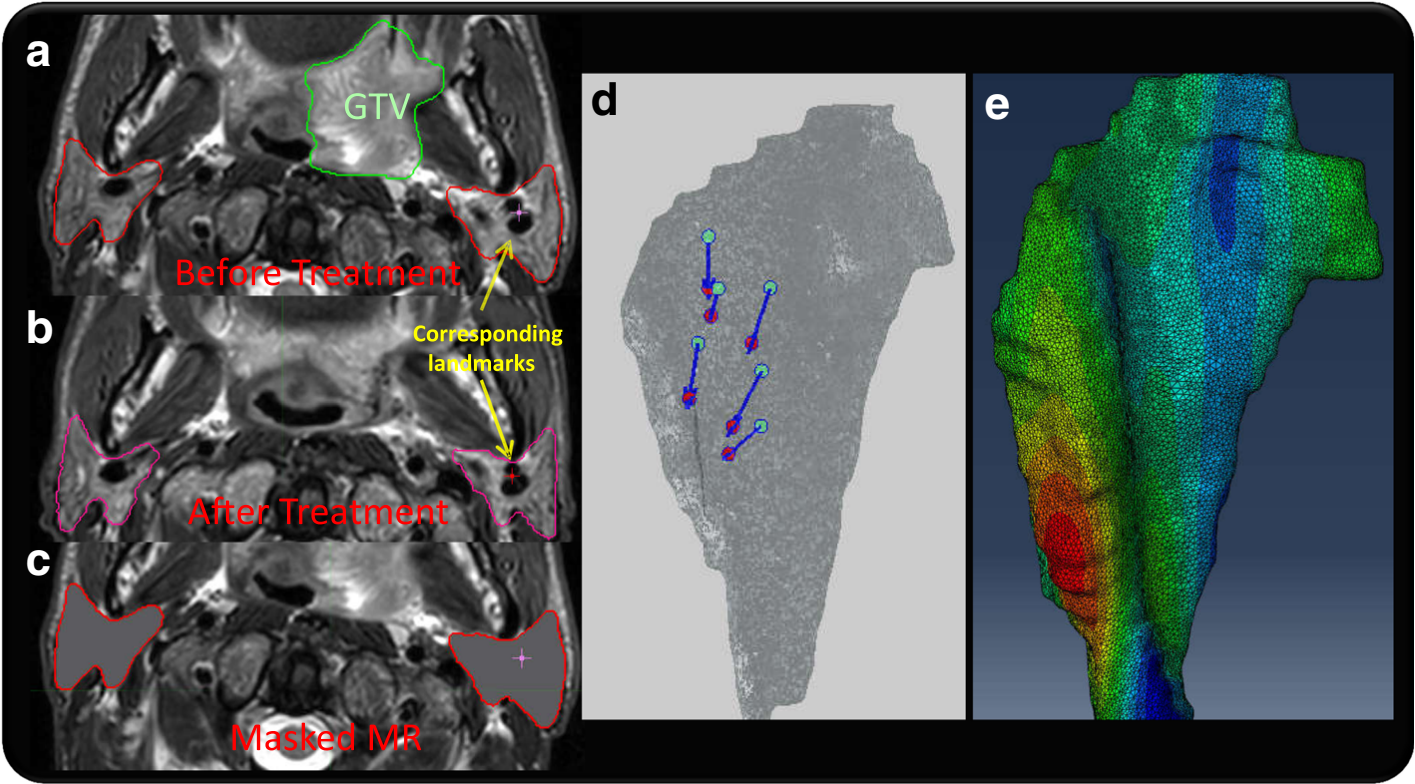
The MR T2w images of Patient#1: **a** Before treatment; **b** After treatment; **c** Parotid-masked image (**d**) Vector from pre-treatment (cyan) to post-treatment (red) landmarks (**e**) Volume mesh with the color rendering of displacement magnitude

Two pairs of MR images (Philips Ingenia 3.0 T, T2W, TR 5187 ms, TE 80 ms) of Head Neck cancer patients have been acquired on two patients before and after radiotherapy under approval from our institutional review board, with the resolution of 0.045, 0.045, 0.306 cm in anterior-posterior, lateral and inferior-posterior direction respectively. The two patients were selected for the parotid shrinkage after radiotherapy and the availability of high-resolution MR with clearly defined internal structure for landmark selection. The parotid contours were delineated on all MR images. In addition, for each parotid, six anatomic landmarks were identified based on the bifurcation of artery and vein as pointed on Fig. 4a, b. The contours and landmarks were reviewed and edited by an experienced radiation oncologist. Most landmarks are located close to the central part of parotids. Figure 4 shows the MR image before (Fig. 4a) and after (Fig. 4b) treatment of patient#1, with physician edited contours. The Gross Target Volume (GTV) in green contour, which was shown on the pre-treatment MR, almost disappears completely on the after-treatment image, which leads to large local deformation. The landmarks of patient#1 are visualized in Fig. 4d with parotid 3D-surface, showing the displacement between two images. The corresponding parotid FEM model with the color rendering of magnitude of DVF is illustrated in Fig. 4e. Similar to the masked phantom MR images, a new set of parotid-masked MR images were generated by overriding the intensity inside parotid to simulate imaging modalities without internal contrast like CT (Fig. 4c). IM-DIR and BM-DIR were performed on both MR and masked MR pairs using pre-treatment image as reference. The target registration error (TRE) was evaluated for the output DVFs based on the expert landmarks.

## Results

**Fig. 5:**
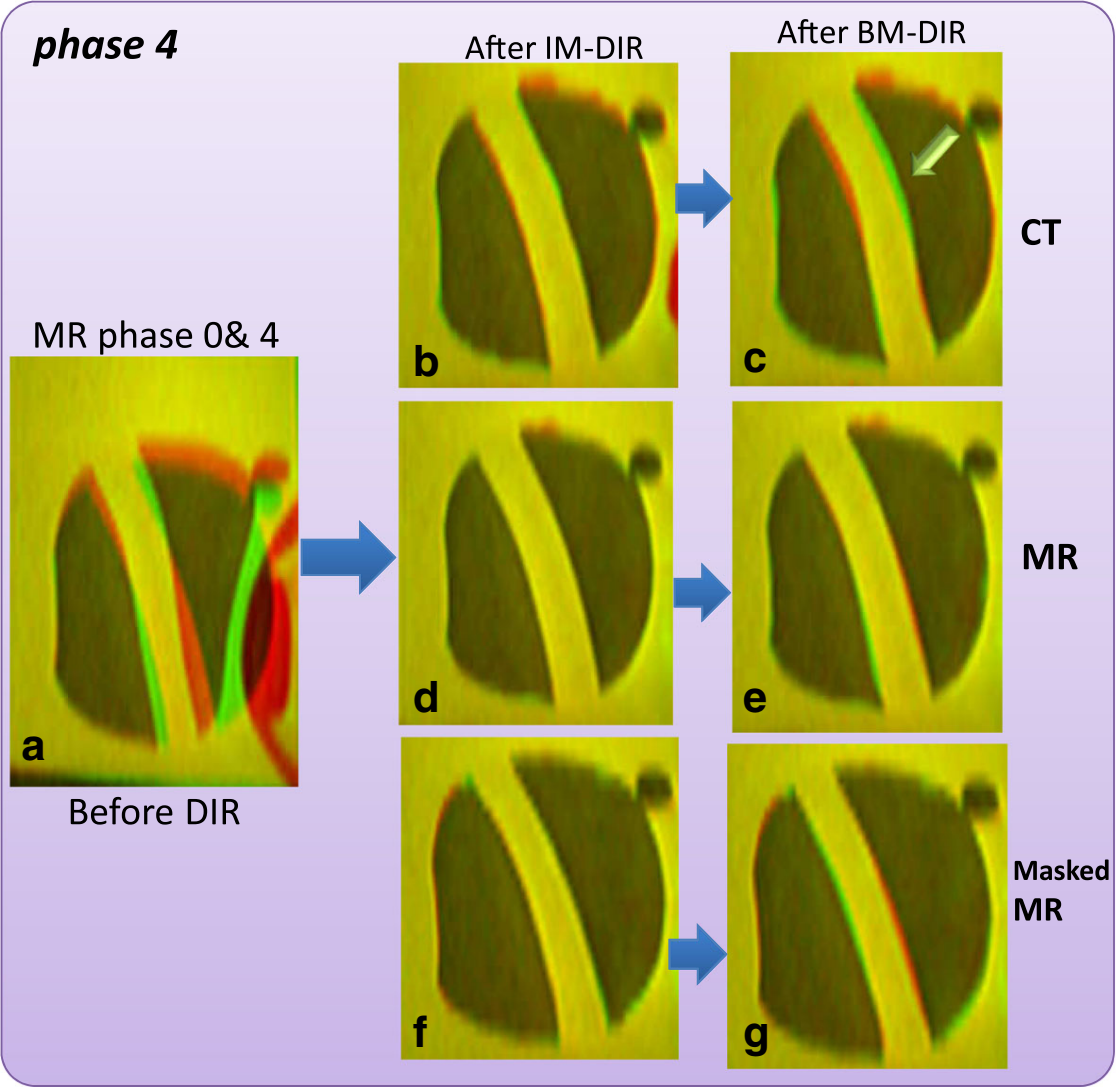
Image fusion for DIR between Phase 0 & 4: **a** before DIR; IM-DIR with (**b**) CT, **d** MR, **f** Masked MR; BM-DIR with (**c**) CT, **e** MR, **g** Masked MR; The green arrow in (**c**) points to the sub-optimal matching of urethra structure after BM-DIR

**Fig. 6:**
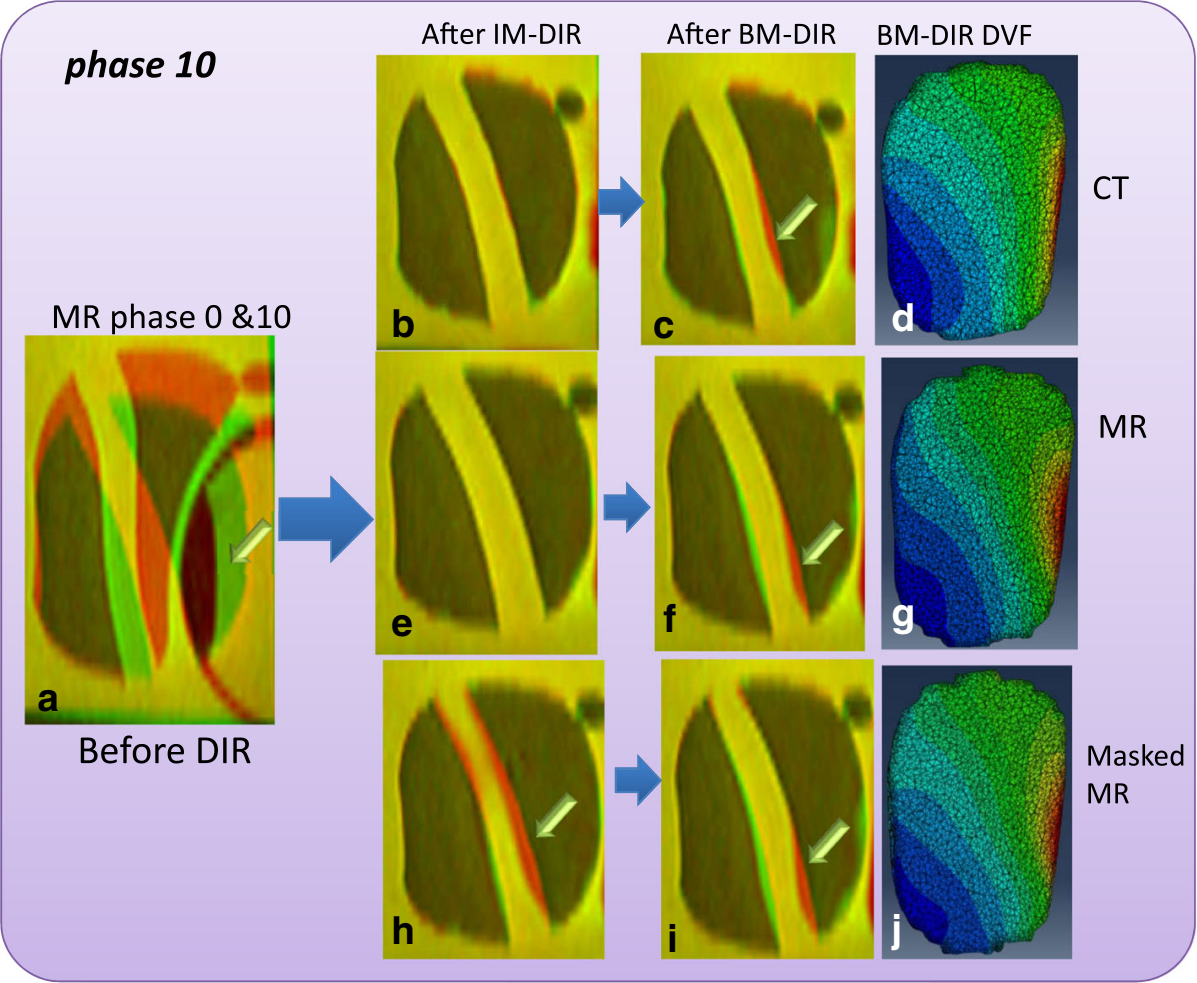
Image fusion for DIR between Phase 0 & 10: **a** before DIR; IM-DIR with (**b**) CT, **e** MR, **h** Masked MR; BM-DIR with (**c**) CT, **f** MR, **i** Masked MR; color rendering motion magnitude of (**d**) CT, **g** MR, **j** Masked MR. The green arrow points to sub-optimal matching of urethra structure after DIR

A volume mesh with 83,119 nodes and 393,089 tetrahedrons was constructed to represent the prostate phantom (Fig. 3c). Both IM-DIR and BM-DIR were applied without user interaction. A same set of prostate contour delineated from MR images were used to constrain the IM-DIR. IM-DIR was able to match the prostate boundary for all three types of images and under all deformation stages. The average computation time on phantom data is less than 1 min for the initial IM-DIR and about 4 min additional for the BM-DIR refinement. The fusion images before, after IM-DIR, and after BM-DIR for phase 4 are shown using the sagittal perspective. The top right row (Fig. 5b, c) shows the warped MR images by using DVF calculated from CT images. Visual inspection of the overlapping urethra tube shows that IM-DIR between CT performed better than the BM-DIR as pointed out by the green arrow in Fig. 5c. For other two types of image (Fig. 5d, e for MR; Fig. 5f, g for Masked MR), there are minor visual differences between IM-DIR and BM-DIR. The fusion images of phase 10 were shown in Fig. 6 where under the extreme deformation, IM-DIR between CT and MR was still able to match the organ surface and the urethra structure (Fig. 6b, e), while small mismatch can be observed for BM-DIR (Fig. 6c, f). However, when using masked MR images (lacking internal distinctive features by design) the IM-DIR failed to match the urethra structure by a relatively large discrepancy as pointed by arrow (Fig. 6h). Using the refined BM-DIR technique, the urethra structure overlap shows less discrepancy than when using the IM-DIR technique (Fig. 6i). The DVF magnitude obtained using the BM-DIR of phase 10 from all three image types is shown in Fig. 6(d, g, j) where the distribution pattern is similar and independent of image types.

**Fig. 7:**
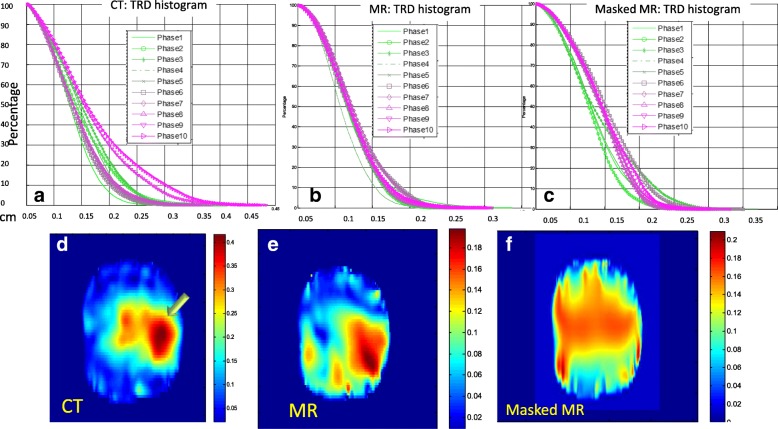
Target Registration Discrepancy (TRD) between IM-DIR and BM-DIR on: **a** 10 phases for CT, **b** 10 phases of MR and (**c**) masked MR; Sagittal view of TRD map on phase#10 for (d)CT, **e** MR and (**f**)Masked MR

The cumulative TRD histogram between IM-DIR and BM-DIR on CT, MR and masked MR images inside the prostate is shown in Fig. 7. As expected, the discrepancy increases with the degree of deformation improving from phase 1 to phase10. For CT DIR, as shown in Fig. 7a, for most phases (1 to 7), less than 6% of the voxels have larger than 0.2 cm (voxel size) distance discrepancy in contrast to phase 10 where the number of voxels with large discrepancies is close to 20%. The spatial distribution map of TRD magnitude for the CT phase 10 is shown in Fig. 7d. Evidently, the biggest discrepancy occurs in regions undergoing the largest deformation as pointed by the arrow in Fig. 6a and Fig. 7d.

Similarly, the cumulative TRD histograms were plotted for MR and masked MR images (Fig. 7b and c respectively). Phase 10 spatial distribution maps of TRD magnitude for MR and masked MR are depicted in Fig. 7e and f, respectively. The discrepancy between MR IM-DIR and BM-DIR is very small when compared with CT, as indicated by the histogram and DVF difference map. The consistency suggests that the IM-DIR algorithm can predict the deformation quite well via MR images which have clear boundary and sharp, well defined internal contrast. After removing the internal contrast, the difference between the two DIR methods increases dramatically. The large discrepancy spreads to wider region as shown by Fig. 7e, f. For the MR image DIR, only 3.5 ± 1.8% (range 1.3–6.0%) of voxels have larger than 0.15 cm TRD. By contrast, the percentages are 17.5 ± 7.9% (6.3–32.7%) and 11.4 ± 4.8% (4.0–18.9%) for CT and masked MR, respectively. There is a significant difference for CT/MR (*p* < 0.001) and MR/MR_masked (*p* < 0.001) in term of percentage of voxel with TRD larger than 0.15 cm, while there is no significant difference for CT/MR_masked (*p* = 0.068). The inferior contrast of CT across the prostate boundary as compared to the masked MR may have caused its larger discrepancy.

**Fig. 8:**
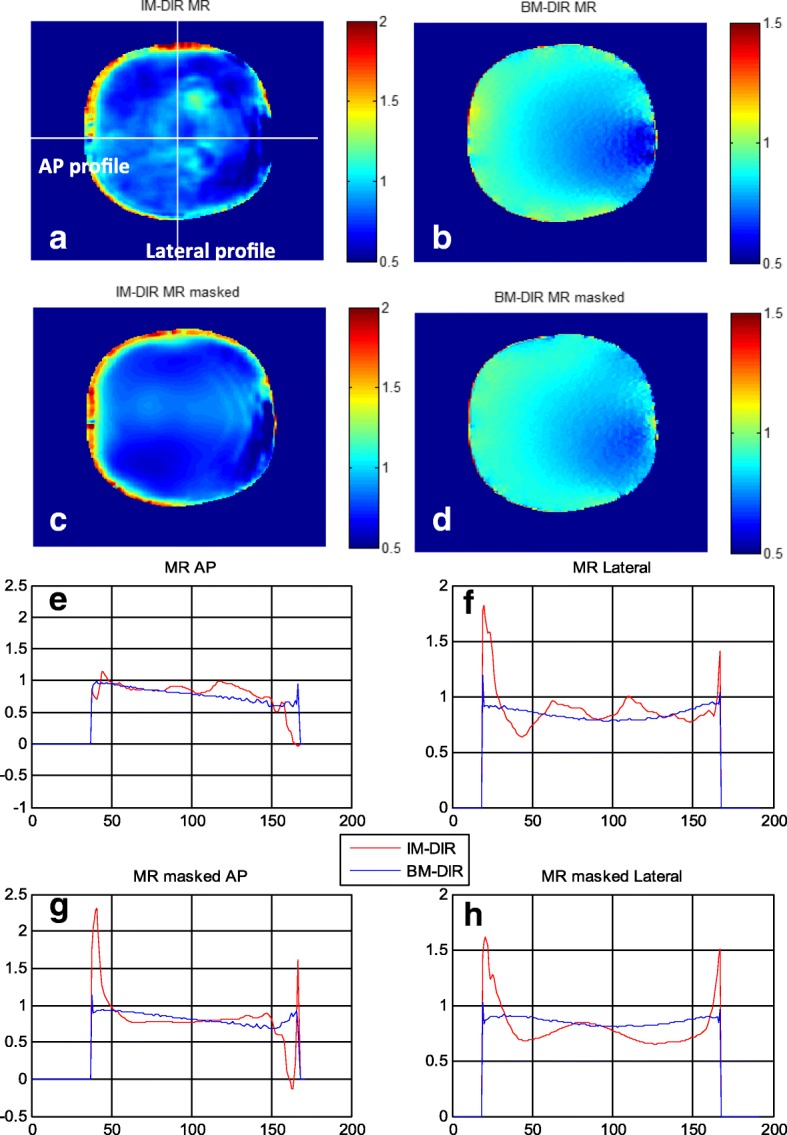
Jacobian map on the central axial slice from DIR between phase 10 and phase 0: **a** IM-DIR on MR, **b** BM-DIR on MR, **c** IM-DIR on Masked MR, **d** BM-DIR on Masked MR. Jacobian profile comparison between IM-DIR and BM-DIR in Anterior-Posterior (AP) and lateral direction: **e** MR AP, **f** MR lateral, **g** MR masked AP, **h** MR masked lateral

**Fig. 9:**
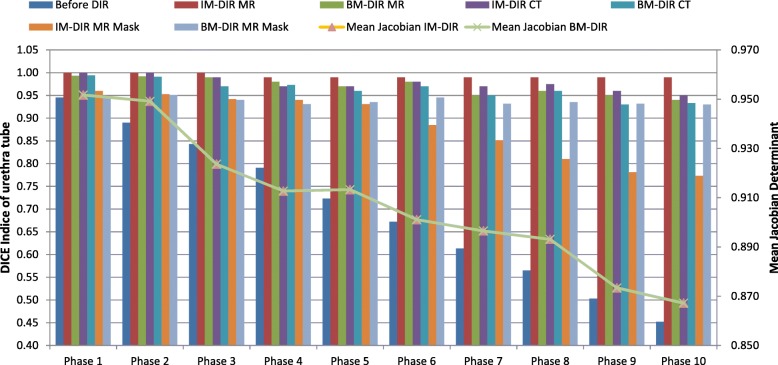
The DICE index (left axis) of the urethra tube before and after DIR; the mean Jacobian determinant (right axis) inside prostate of all 10 phases for IM-DIR and BM-DIR

To inspect the physical plausibility of the DVFs, the Jacobian determinant maps of the central axial slice and its corresponding profiles, calculated from the phase-10 DVFs of the MR and masked MR phantom images, are shown in Fig. 8. Jacobian value less than 1 means contraction and larger than 1 means dilation. As shown in the Jacobian map of MR (Fig. 8a) and masked MR (Fig. 8c), there exists significant irregularity of contraction/expansion inside prostate especially near the boundary. In contrast, the Jacobian maps of BM-DIR are much smoother for both MR (Fig. 8b) and masked MR (Fig. 8d) with similar pattern. The profiles extracted in both anterior-posterior and lateral directions give a closer view of the Jacobian comparison (Fig. 8e, h). As expected, the Jacobian profiles of IM-DIR show more significant variation than those of BM-DIR. The mean Jacobian values of both DIR methods inside the prostate are plotted in Fig. 9, which agree quite well with each other, showing a continuously decrease from 0.95 to 0.87 from phase 1 to phase 10.

**Fig. 10:**
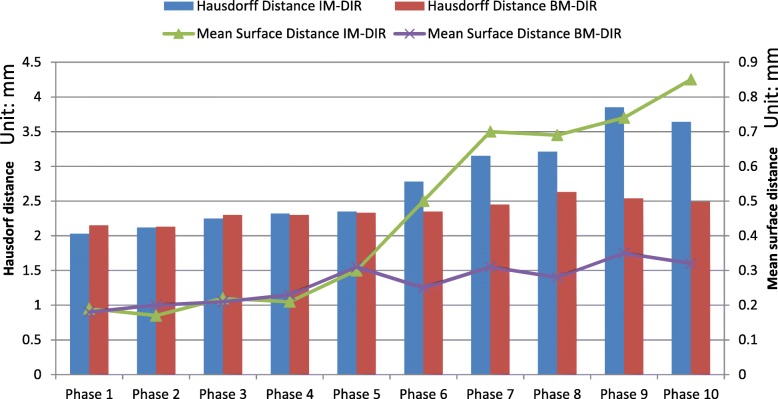
The Hausdorff and mean surface distance of the urethra tube after IM-DIR and BM-DIR on MR mask images

The DSC of the urethra tube-- before and after DIR—is used as an indicator of internal deformation accuracy and illustrated in Fig. 9. The DSC between un-deformed phase and subsequent deformed phases of the original images decreases from 0.95 (phase 1) to 0.45 (phase 10). Using the CT and MR images, IM-DIR was able to achieve > 0.95 overlap for all phases (MR: 0.99 ± 0.005, CT: 0.98 ± 0.016). The DSC of BM-DIR on original CT and MR were slightly lower (MR: 0.97 ± 0.019, CT: 0.96 ± 0.022) when compared with IM-DIR. However, for masked MR, BM-DIR (0.94 ± 0.008) is significantly better than IM-DIR (0.88 ± 0.074, *p* < 0.001), especially for phases of large deformation (phase 6 to 9). For example, BM-DIR was able to achieve 0.93 in phase 10 of masked MR, while IM-DIR got only 0.77. The fusion images (Fig. 6h, i) also demonstrated a better urethra tube overlapping when using BM-DIR for phase 10. Figure 10 show the Hausdorff as well the mean surface distance of the urethra tube after both DIR methods on the masked MR images. Similar to DSC, the BM-DIR outperforms the IM-DIR in term of both surface distance measures starting from phase 6.

**Fig. 11:**
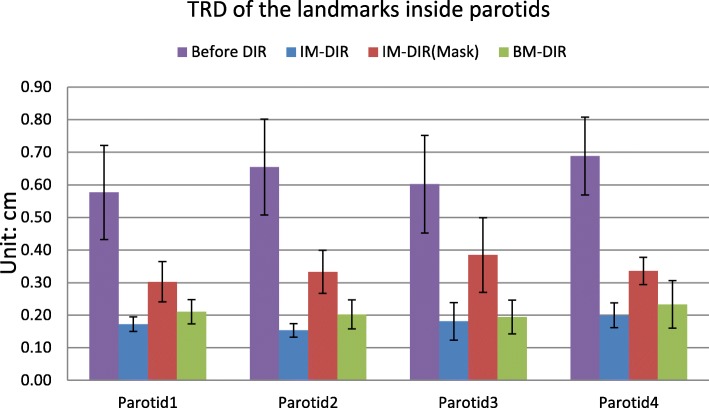
The TRE of the landmarks inside parotid before and after two types of DIR for two Head&Neck patients

The volume meshes of patient parotids include 109,916 nodes and 627,408 tetrahedrons on average. The volumes of parotids shrink by 20.4 ± 5.9% (mean ± STD) after treatment (ranging from 14.9 to 26.8%), which is relatively small compared to median volume loss of 28.1% reported in [45]. The TRDs of three types of DIR on four parotids are illustrated in Fig. 11.The IM-DIR on original MR was able to register the parotid surfaces and internal structure quite well, due to the excellent contrast on the MR images. On average, the displacement of landmarks is 0.630 ± 0.14 cm before DIR, 0.177 ± 0.03 cm after IM-DIR on original MR and 0.339 ± 0.07 cm after IM-DIR on masked MR. By the BM-DIR refinement, the TRE of landmarks were improved to 0.210 ± 0.05 cm on the masked MR image.

## Discussion

In this study, we have developed a biomechanical DIR refinement method and evaluated it on a deformable tissue-equivalent phantom with reproducible degree of deformation. Using the internal urethra structure as a surrogate, we demonstrated an increased robustness of BM-DIR when large deformation is present and there is lack of distinctive image features. It should be noted that only surface nodes displacement was used in the BM-DIR to simulate the internal deformation. The urethra tube inside is not explicitly meshed, modelled or included in the boundary condition, in order to simulate intensity homogeneous soft tissue organs on CT images. The less optimal DSC and surface distance, as well as relative large TRD by IM-DIR on the masked MR images of large deformation (phase# > 6, DSC < 0.67, average Jacobian< 0.9) implies the BM-DIR refinement has the potential to improve DIR accuracy of the organ of interest with large deformation and where little or no image feature is present.

To the best our knowledge, this is the first study that used a phantom with multi-stage deformation to evaluate potential DIR accuracy improvement by biomechanical model. Based on our evaluation, for solid organs that undergo large deformation (DSC less than 0.67 and mean Jacobian less than 0.9), the proposed hybrid BM-DIR method could improve the accuracy and physical plausibility of the internal DVF inside intensity-homogeneous organ. The same potential improvement may be taken advantage of for image modalities with small contrast to noise ratio(e.g. CBCT, ultrasound), where little to no matching image feature exist inside soft tissue organs or for cross-modality DIR (CT-US, CT-CBCT, CT-MR).

One of the limitations of this study is that the phantom may not be able to sufficiently approximate the complex deformation of human organ. We have demonstrated the improvement of DIR accuracy on masked H&N MR images inside parotid for two patients. However, a more comprehensive evaluation including larger patient data set, various treatment sites and imaging modalities are warranted in the future to confirm the validity of the proposed hybrid methodology, which is beyond the scope of this study. The quality of DIR could also be improved by adding special engineered regularization terms, constrains of the Jacobian of the deformation or its derivatives [46]. These terms are usually designed for specific applications and integrated in the DIR algorithm.

For organs with distinctive image features/contrast inside like lung and bone, state of art IM-DIR algorithms can achieve high accuracy which could be easily evaluated and validated by visible anatomical landmarks. However, for intensity homogeneous organs, the internal DVF will depend mostly on regularization terms, which are often non-physical and varied significantly between different algorithm implementations and commercial vendors. For example, the less smooth Jacobian profile of IM-DIR as shown in Fig. 8 may be explained by the limited range that the regularization term can propagate the deformation from the boundary to the inside intensity-homogeneous region. By contrast, the proposed hybrid-DIR is an independent tool to refine the output of any clinical commercial DIR tools.

The focus of this study is to quantitatively demonstrate the benefit of the hybrid BM-DIR on the multi-stage deformable phantom. Various hybrid or pure FEM model based DIR methodologies have been proposed previously. For example, the well-known MORFEUS method [22] is based on a multi-organ pure FEM model. The surface boundary conditions were assigned by the surface matching based on the geometric characteristic without utilizing image information. Recently, a hybrid biomechanical model was proposed to improve the accuracy of the original MORFEUS for lung CT by incorporating intensity-based DIR after the physical model-based DIR [47]. FEM methods have also been proposed to correct the intensity based DIR in low contrast regions for CT [23] and for CT/MR DIR for prostate patient [24]. In their studies, the boundary conditions were also assigned by the initial intensity-based DIR either on high-contrast regions or on a bounding box of organ of interest.

As proposed in the recent published AAPM report task group 132 (TG 132) [48], a well-documented patient-specific verification is essential for DIR quality assurance after the initial commissioning of any DIR tools. In clinical routine, accurate landmarks are often either very difficult to define (for example, inside CT prostate or parotid) or too time consuming to be practical, or sometimes unnecessary since modern IM-DIR algorithms can match those high-contrast landmarks with lower than human uncertainty (for example, CT lung) [49]. Therefore, the large discrepancy between BM-DIR and IM-DIR on intensity-homogeneous region could potentially serve as a trigger for further investigation of the physical plausibility and accuracy of DIR, especially for advanced applications like dose wrapping. The BM-DIR described here can be executed automatically following any intensity-based DIR. If large deviation is observed between these two methods, carefully inspection of the DVF may be necessary before using it for other applications, such as dose warping.

Future improvement of our BM-DIR method will be focusing on incorporating automatic detected distinctive internal features as an additional boundary condition (e.g. N-SIFT feature [50]). To speed up FEM calculation and better model soft tissue deformation, GPU accelerated FEM calculation by open source packages like NiftySim [51] and its nonlinear model is also being evaluated to replace the commercial FEM tool we current use.

## Conclusion

Biomechanical model based DIR refinement could be beneficial for improving voxel-wise accuracy in the absence of internal distinctive image feature, especial for organ with large deformation.

## Abbreviations

ART: adaptive radiation therapy
BM-DIR: biomechanical-model based DIR
CT: Computed Tomography imaging
DIR: Deformable Image Registration
DSC: Dice similarity coefficient
DVF: deformable vector field
FEM: Finite-Element method
GTV: Gross Target Volume
IM-DIR: Intensity-based DIR
LCC: local-correlation-coefficient
MR: Magnetic Resonance imaging
TRD: Target Registration Discrepancy
TRE: target registration error
